# Ribosome Specialization in Protozoa Parasites

**DOI:** 10.3390/ijms24087484

**Published:** 2023-04-19

**Authors:** Cristian Camilo Rodríguez-Almonacid, Morgana K. Kellogg, Andrey L. Karamyshev, Zemfira N. Karamysheva

**Affiliations:** 1 Department of Biological Sciences, Texas Tech University, Lubbock, TX 79409, USA; 2 Department of Cell Biology and Biochemistry, Texas Tech University Health Sciences Center, Lubbock, TX 79430, USA; morgana.kellogg@ttuhsc.edu (M.K.K.);

**Keywords:** ribosome heterogeneity, ribosome specialization, ribosomal RNA, ribosomal protein, mRNA translation, translational control, Trypanosomatidae, Apicomplexa

## Abstract

Ribosomes, in general, are viewed as constitutive macromolecular machines where protein synthesis takes place; however, this view has been recently challenged, supporting the hypothesis of ribosome specialization and opening a completely new field of research. Recent studies have demonstrated that ribosomes are heterogenous in their nature and can provide another layer of gene expression control by regulating translation. Heterogeneities in ribosomal RNA and ribosomal proteins that compose them favor the selective translation of different sub-pools of mRNAs and functional specialization. In recent years, the heterogeneity and specialization of ribosomes have been widely reported in different eukaryotic study models; however, few reports on this topic have been made on protozoa and even less on protozoa parasites of medical importance. This review analyzes heterogeneities of ribosomes in protozoa parasites highlighting the specialization in their functions and their importance in parasitism, in the transition between stages in their life cycle, in the change of host and in response to environmental conditions.

## 1. Introduction

Ribosomes translate the genetic information encoded in mRNAs into functional polypeptides and proteins. Ribosomes are complex structures, consisting of 4 ribosomal RNAs (rRNAs) and 80 ribosomal proteins (RP) in eukaryotes (79 in *Saccharomyces cerevisiae*) [1], forming 2 subunits: a small subunit (40S), composed of 18S rRNA and 33 RPs, and a large subunit (60S), constituted by 28S, 5.8S, and 5S rRNAs and 47 RPs [1,2].

The ribosome has been considered highly conserved within species, which is why 18S rDNA sequences are commonly used for phylogenetic reconstructions [3]. However, recent studies have revealed that ribosomes are not invariant and homogeneous entities, on the contrary, they show different types of heterogeneity [4]. Despite ribosome heterogeneity being reported for the first time in *Escherichia coli* in the 1970s [5,6], with subsequent reports of ribosome variability and specialization in the 1980s and 1990s [7,8,9], it was only after the postulation of “the ribosome filter hypothesis” in 2002, that the premise that ribosome heterogeneity can lead to specific interactions between ribosomes and mRNAs, triggering differential rates in mRNA translation [10,11], was established. Later studies have shown that heterogenous ribosomes display functional specialization, which boosts the translation of selective sub-pools of mRNAs [12,13,14,15,16], supporting “the ribosome filter hypothesis”. Although recent data support the theory of ribosome specialization in mammals [17], the data validating the ribosome specialization and its functional role in other organisms including protozoa are very limited; despite this, the ribosome heterogeneity in these organisms is undeniable. Likewise, it has been demonstrated that ribosomes expressing these heterogeneities are essential for proper differentiation of stem cells and embryo development [18], vertebrate tissue patterning [19], and even, could have a predominant expression in some tissues, such as testis [20,21,22,23,24,25,26], or as a response to stress [27,28]. This evidence highlights ribosomes as active molecules involved in translational control, further from their static role as molecular machines with the only purpose of protein synthesis.

Currently, it remains unclear how specialized ribosomes are formed. It is possible that specialized ribosomes could be synthesized de novo or could be remodeled through the exchange of the ribosomal components. The first scenario is likely to occur in the case of ribosome heterogeneity in different tissues in mammals when certain paralogs are dom-inant. In the second scenario, specialization may involve the remodeling of existing ribosomes where the core structure of the ribosome will be reused, and the surface proteins are replaced with de novo synthesized paralogs. The second scenario is more likely to be used when very fast changes are required, for example, during stress. In this case, it is likely only proteins close to the ribosome surface can be exchanged or modified directly in the cytoplasm. Some data indeed support that ribosome composition is not fixed after biogenesis and exchange of RPs with mature ribosomes may occur. However, overall, these extremely interesting questions about two scenarios are very difficult to address experimentally and require further investigation.

### 1.1. Types of Ribosome Heterogeneity

Several studies have reviewed the types of ribosome heterogeneity observed in different organisms, which can be classified into three categories according to their origin: ribosomal protein heterogeneity, ribosomal RNA heterogeneity, and heterogeneity by ribosome-associated factors [4,29,30,31,32,33,34,35,36].

A common type of RP heterogeneity is related to the presence of multiple paralog genes encoding them, as in the case of *S. cerevisiae*, where 59 of 79 RPs are encoded by duplicated genes, many of them with no functional redundancy [37]. An example of this specialization was observed in uL30 genes, where the uL30A paralog (hypoacetylated) is predominantly translated in natural conditions and is required for optimal ribosomal biogenesis and cell growth; however, when cells were exposed to staurosporine, uL30A translation was inhibited and uL30B (hyperacetylated) increased its translation rate, causing the yeasts’ resistance to the drug [28]. Some paralogs have also shown differential expression and functional specialization in some tissues such as mouse’s testis, where RPL39L, the paralog of ribosomal protein RPL39/eL39, is mainly expressed later in spermatogenesis and is essential for fertility in mice [26]. On the other hand, RPs can be also heterogeneous in stoichiometry, which means, they have a variation in their equimolar proportions. This has been observed in mouse embryonic stem cells (mESCs), where 6 of 15 assessed RPs showed sub-stoichiometric measurements, and 4 of them were in 60% to 70% of polysomal ribosomes, indicating that translating ribosomes lack at least one RP [12]. Ribosomal proteins have also shown variability by post-translational modifications, including methylation, ubiquitination, acetylation, and phosphorylation. An example of this was observed in different cell culture lines, where phosphorylation of Serin 38 (S38) in RPL12/uL11 boosted the association of phosphorylated ribosomes with mRNAs actively translated during mitosis [38]. Likewise, in the stationary growth phase of yeast, a 10-fold increase in dimethylation of arginine 10 (R10) in RPS2/uS5, slowing down the processing and export of rRNA 20S, was observed as a possible response to nutrient depletion [39].

Ribosomal RNA (rRNA) heterogeneity can be caused by changes in rDNA alleles and their variable copy number across individuals. This was observed in mouse and human sequences, where intra and inter-individual nucleotide sequence heterogeneities were identified in all four rRNA genes, as well as tissue-specific expression of rRNA variants [40]. Additionally, rRNA can exhibit three different types of post-transcriptional modifications in nucleotides [41]: conversion of uridine to pseudouridine (Ψ) [42,43], methylation of ribose’s 2′ hydroxyls (2′-O-methylations) [44,45], and methylation of bases [46]. These modifications are guided by small nucleolar RNAs (snoRNAs) and stand-alone enzymes and are important for translational initiation and fidelity, stability of ribosome structure and biogenesis [47,48].

In addition to variations in rRNAs and RPs, ribosome-associated factors also serve as a source of ribosome heterogeneity and specialization. These factors are proteins binding to the ribosome that mediate certain processes in translation. One of them is the Receptor for Activated C Kinase 1 (RACK1) which is essential for the full and efficient translation of capped mRNAs and efficient recruitment of eukaryotic initiation factor 4E (eIF4E) [49,50]; its depletion or mutation alters the translation of specific mRNAs and can cause induction of autophagy [50]. The Fragile X Mental Retardation Protein (FMRP) is a polysome-associated RNA-binding protein regulating neuronal translation. It stalls ribosome translocation during elongation and serves as a translational brake on a selective set of transcripts encoding synaptic proteins. Loss of FMRP leads to Fragile X syndrome (FXS) and autism [51].

In addition to all these types of ribosome heterogeneity reported in cytosolic ribosomes, mitochondrial ribosomes (mitoribosomes) have undergone substantial changes during their evolution, differing from bacterial ribosomes with respect to composition and structure [52,53]. The changes include the acquisition of numerous ribosomal proteins, some of which are exclusive to mitochondria, and reduce the rRNA content in half compared to bacteria’s ribosomes (Table 1) [54,55,56,57,58,59]. As a result, mitoribosomes exceed the bacterial ribosomes in molecular mass and physical dimensions; additionally, they have a strong functional specialization, synthetizing exclusively hydrophobic membrane proteins in mammals, as well as mitochondrial ribosomal subunits, enzymes, and assembly factors in other kingdoms [60].

### 1.2. Ribosome Heterogeneity and Specialization in Non-Parasitic Protozoa

**Figure 1:**
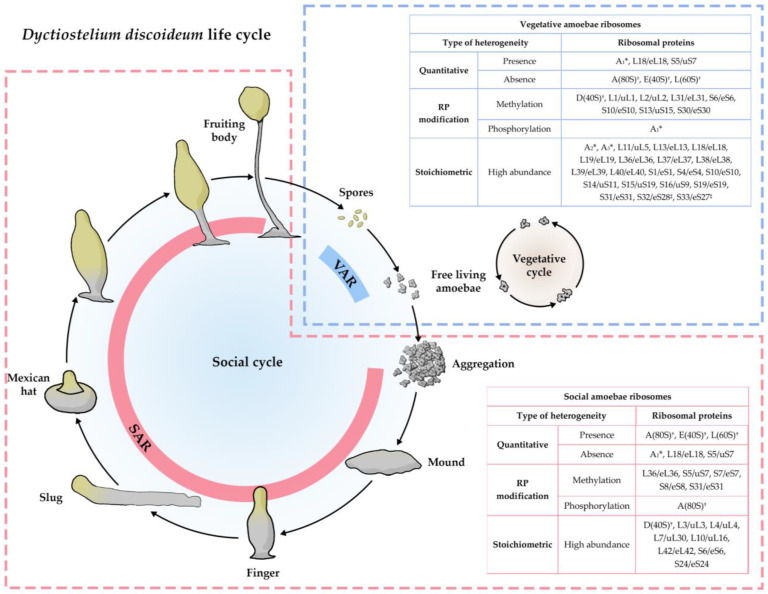
Ribosome switching in *Dyctiostelium discoideum’s* life cycle. The vegetative cycle of *D. discoideum* (light blue dotted box) includes free-living amoebae that reproduce by binary fission when there are proper nutritional conditions; in this cycle, vegetative amoebae ribosomes (VAR) express the ribosomal proteins (RP) shown in the blue table. The social cycle (light red dotted box) starts when there are deficient nutritional conditions, forcing amoebae to aggregate and form a multicellular organism. Social amoebae ribosomes (SAR) express a different set of RPs and modification patterns, which are shown in the light red box. The correlation of the new nomenclature was conducted following the new system for naming ribosomal proteins [73]. * The correlation of these proteins with the current nomenclature is unknown. † These proteins are known to be associated with the respective ribosomal subunits, but no numerical designation was given by the authors [69]. ‡ Correlation of these proteins were made, taking into account the correlation for yeast ribosomal proteins made by Moore (1991) [74].

One of the first and most studied protozoans where ribosome heterogeneity and specialization were reported is *Dictyostelium discoideum*, a soil-dwelling social amoeba. This organism lives in soil and feeds on bacteria; however, starvation induces amoebae aggregation and it becomes a multicellular organism that undergoes cell differentiation and morphogenesis, to finally produce the fruiting body that comprises a spore-containing sorus resting upon a stalk [67,68] (Figure 1).

Developmental regulation of RPs has been described in *D. discoideum,* where different populations of ribosomes are assembled in distinct stages of their life cycle [69,70,71]. The change in ribosome composition takes place in two phases, the first one (activation) occurs after nutrient starvation, leading to the development of a different population of ribosomes that accumulate in the fruiting body (social amoebae ribosomes—SAR); the second phase (deactivation) occurs during the spores’ germination, causing an accumulation of a different set of ribosomes in vegetative amoebae (vegetative amoebae ribosomes—VAR) [72]. The RP heterogeneity observed in *D. discoideum* has been classified into three categories: quantitative, covalent modifications, and stoichiometric, according to its presence or occurrence in vegetative amoebae and developing cells or spores. The first one includes the presence or absence of certain RPs in both stages; the covalent modifications encompass modifications of RP, such as methylations and phosphorylations and, finally, stoichiometric heterogeneity is reported as a rise in the abundance of some RP in one stage compared to the other [72] (Figure 1).

In addition to *D. discoideum*, there are several reports about ribosome heterogeneity and specialization in other organisms [4,29,31,35,36]. However, despite the raising importance of these variabilities in regulation of gene expression at the translational level, scarce information is available about it for protozoa parasites. Most of these parasites are characterized by switching between two or more hosts, facing challenging environments, and developing elaborated mechanisms to survive, of which ribosomes may assume important functions. In this review, we focused on the role of ribosome heterogeneity and specialization in the biology of digenetic protozoa parasites of medical interest, with special emphasis on *Plasmodium* spp., *Trypanosoma* spp., and *Leishmania* spp.; these parasites represent excellent models to study ribosome specialization due to their digenetic nature of the life cycle.

## 2. Ribosome Specialization in *Plasmodium* spp.

**Figure 2:**
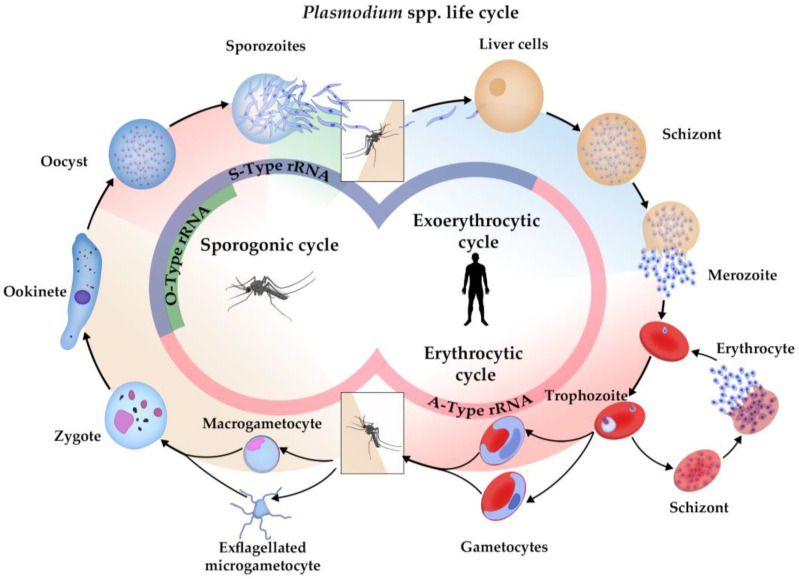
Types of SSU ribosomal RNA expressed in *Plasmodium* spp. life cycle. A-type rRNA (the red inner circle) is transcribed early in the exoerythrocytic cycle of *Plasmodium* in liver cells of a vertebrate host, being dominant until the zygote stage in the midgut of a mosquito. S-type rRNA (blue inner circle) expression starts in zygotes and is maximally transcribed in the sporozoite stage at salivary glands of mosquitoes; being reduced after infection of liver cells. The O-type rDNA (green inner semicircle) was only described in *P. vivax*, which is expressed in maturing ookinetes and oocysts and was not detectable in sporozoites from mosquitoes.

*Plasmodium* genera is composed of apicomplexan parasites, which include *P. falciparum* and *P. vivax*, the main etiological agents of malaria in humans. The life cycle of this parasite includes two hosts, the *Anopheles* vector mosquitoes in which the sporogonic cycle occurs, and the vertebrate host, where the erythrocytic and exoerythrocytic phases take place (Figure 2). In exoerythrocytic phase, parasites multiply asexually in liver cells before differentiating into merozoites and then enter into the bloodstream and initiate erythrocytic phase. Each stage has a peculiar morphology and physiology that is determined by its gene expression profile, which is carried out by epigenetic, transcriptional, post-transcriptional, and post-translational regulatory mechanisms [75].

Unlike other eukaryotes, in which rDNA is composed of hundreds of tandem repeated units, *Plasmodium* has 4–8 single-copy rDNA units distributed in different chromosomes [76]. rDNA is the main source of ribosome heterogeneity in *Plasmodium,* evidenced by the presence of different types of rRNA genes, which are structurally distinct and expressed at different stages of the *Plasmodium* life cycle [77,78]. The small subunit rRNA (SSU rRNA) transcripts from A-type (asexual) gene predominate in hepatic and bloodstream stages, while S-type (sporozoite) gene transcripts are prevalent in mature sporozoites; additionally, a third type of rRNA gene (O-type gene) was discovered in *P. vivax,* which is expressed in maturing ookinetes/oocysts [76,79] (Figure 2). Further studies demonstrate that the switching of transcriptional activity between the different rRNA gene types was linked to differentiation pathways and developmental progression in a species-specific manner [80,81].

The functional specialization of ribosomes containing these rRNAs remains controversial. The large subunit rRNA (LSU rRNA) from A- and S-type in *P. falciparum* showed functional differences in chimeric rRNAs experiments [82]. The GTPase domain of the 25S rRNA from *S. cerevisiae* was replaced by GTPase domains from *Plasmodium* A- and S-type. Cells with A-type chimeric rRNA grew normally, but S-type chimeric rRNA was lethal. Additionally, it was demonstrated that one of the S-type genes (D-type SSU) in *P. yoelii*, an etiological agent of rodent malaria, is essential for oocyst and sporozoite development, and disruption of this gene causes the oocyst development defect (OOD), characterized as having small oocysts and lacking infective sporozoites [83]. However, studies made on both types of rRNAs from *P. berghei*, another causative agent of rodent malaria, did not show structural differences in core regions of LSU rRNA molecules including GTPase sites. Moreover, knockout of the S-type gene units did not affect the development of this parasite in the vertebrate and mosquito hosts, which could indicate a functional equivalence of both rRNA genes by a gene dosage phenomenon [84].

On the other hand, *Cryptosporidium parvum*, an intestinal Apicomplexa monogenean protozoa phylogenetically close to *Plasmodium* spp., has also shown heterogeneity in 18S rRNA and the presence of two types of rDNA units: Type A and type B, which differentiate each other from the internal transcriber space regions and their number of copies, being four for type A and one for type B [85,86]. Despite this similarity in ribosome heterogeneity with *Plasmodium* spp., the functional significance of this difference is not known in *C. parvum*.

Ribosome-associated factors contribute to ribosome heterogeneity and regulate translation. Relatively little is known about their role in *Plasmodium* spp.; however, some examples support their importance in these species. RACK1, an important regulatory component of mRNA translation, has been found to be associated with 80S and actively translated polysomes in malaria parasites [87].

### Organellar Ribosomes of Plasmodium

*Plasmodium* parasites harbor a single and crucial mitochondrion with a reduced mitochondrial DNA (mtDNA), which encodes two rRNAs and only three proteins: Cytochrome *c* oxidase subunit I (COI), Cytochrome *c* oxidase subunit III (COIII) and Cytochrome *b* (Cyb) [88,89]. Mitoribosomes of *Plasmodium* also have a reduced rRNA content, with 2037 nucleotides in total; however, unlike most of the organisms, this rRNA is highly fragmented, where SSU rRNA is composed of 12 fragments totaling 804 nt, while LSU rRNA is composed 15 fragments totaling 1233 nt [90]. Regarding ribosomal proteins, there is limited available information with up to 43 mitochondrial ribosomal proteins (MRP) identified in *P. falciparum,* based on sequence similarity with known MRP of bacterial and mitochondrial origin; however, the species-specific *Plasmodium* MRP still remains unknown [91,92]. Likewise, *Plasmodium* also harbors the apicoplast, which is a relic of plastids that contains its own DNA and is transcribed by its own apparatus [92]. The apicoplast’s DNA has a complete set of rRNAs and a cluster of ribosomal proteins genes of plastid and bacterial origins [93]. Like mitoribosomes, ribosomes from the apicoplast are also reduced, composed of approximately 40 ribosomal proteins and a large and small rRNA subunit, but these are not fragmented [94].

Despite the limited information available, the importance of some ribosomal proteins has been described. For instance, the knockdown of MRPL13/uL13m caused a deficiency in mitochondrial electron transport chain (mtETC), loss of mitochondrial membrane potential (ΔΨ_m_), hypersensitivity to proguanil and death [95]. Likewise, MRPL12/bL12m, MRPL17/bL17m, MRPL18/uL18m, and MRPL23/ul23m are important for proper enzymatic activity of cytochrome *bc_1_* complex and the resistance to *bc_1_* inhibitors [91,96]. Additionally, the protein ARPS10 from the apicoplast has shown an important role in tolerance to febrile temperatures and artemisinin, showing the role of this plastids in drug resistance [97,98].

## 3. Ribosome Specialization in Trypanosomatids

Trypanosomatidae is a family of parasites taxonomically located in the order Kinetoplastida, which are characterized by having extranuclear DNA known as kinetoplast DNA [99]. This family includes the causative agents of Sleeping sickness disease (*Trypanosoma brucei*), Chagas disease (*T. cruzi*), and Leishmaniasis (*Leishmania* spp.). Trypanosomatids have evolved in different ways than other eukaryotes, developing unique cellular and genetic pathways, such as polycistronic transcription and trans-splicing of pre-mRNAs, leading to a distinctively structured mRNA, which includes the spliced leader (SL), a chain of 39 identical nucleotides at the 5′ terminus with methylation in the first four nucleotides [100,101]. Likewise, ribosomes of Trypanosomatids have shown unique features, such as a 25/28S rRNA fragmented into 6 transcripts, the presence of unusually large expansion segments (ES), and ribosomal protein extensions, as well as additional rRNA insertions, including one exclusively large rRNA domain [102,103,104,105].

### 3.1. Trypanosoma spp.

**Figure 3:**
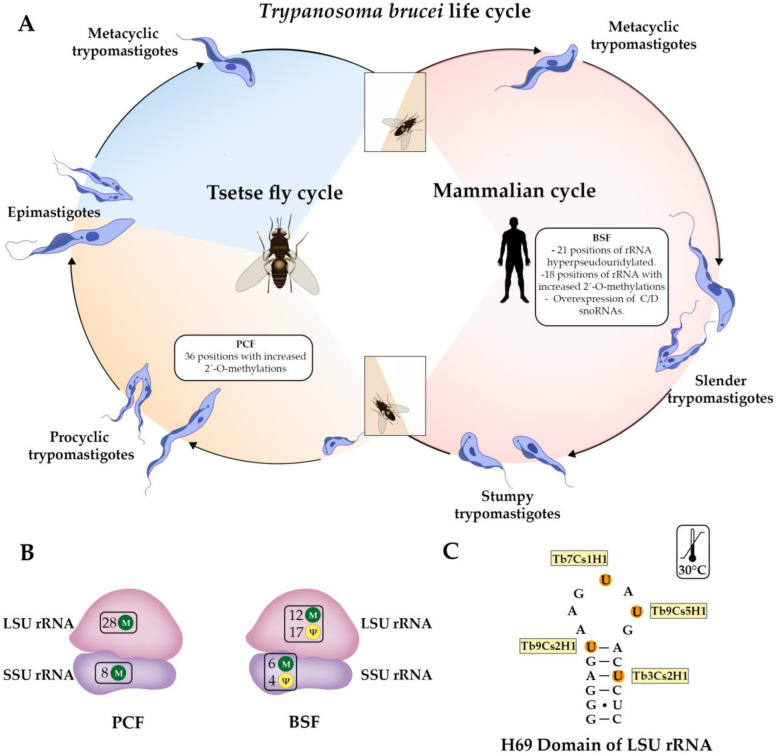
rRNA modifications observed in different stages of the *Trypanosoma brucei* life cycle. (**A**) Bloodstream forms (BSF), mainly represented by slender replicative trypomastigotes in the blood of the mammalian host, show 21 hyper-pseudouridylated (Ψ) positions in rRNA, many of them in domains located in the peptidyl-transferase center (PTC), as well as 18 positions with 2′-O-methylations increased, which is accompanied by the overexpression of most of C/D snoRNAs. Procyclic forms (PCF), also known as procyclic trypomastigotes, display an increase in 2′-O-methylations in 36 positions of rRNA compared with BSF. (**B**) Number of 2′-O-methylations (green circles with an M) and pseudouridylations (yellow circles with Ψ) in the large subunit (LSU) and the small subunit (SSU) of the ribosome in BSF and PCF. (**C**) Hypermodified positions in Helix 69 (H69) of LSU rRNA. The H/ACA snoRNAs involved in the pseudouridylation of ribosomal RNA are highlighted in yellow boxes. Overexpression of these snoRNAs in PCF favors the acceleration of their growth at 30 °C.

**Figure 4:**
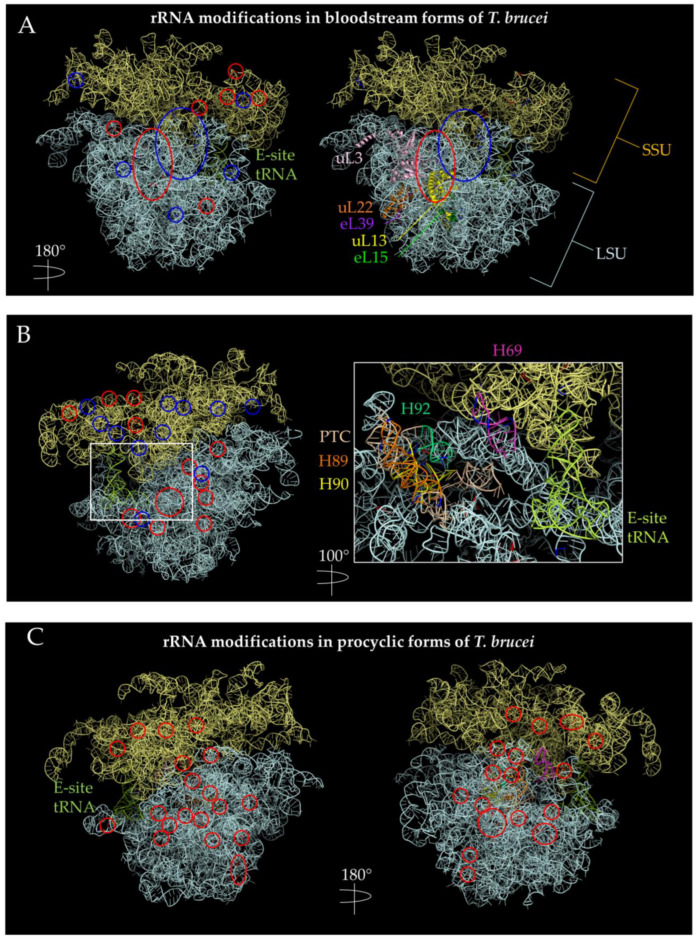
Positions of the rRNA with hyperpseudouridylations and increased 2′-O-methylations in bloodstream forms (BSF) and procyclic forms (PCF) of *T. brucei*. PDB 4V8M [104] ribosome structure were used to visualize methylation and pseudouridylation sites using PyMOL [110]. The SSU is indicated by pale yellow, while the LSU is indicated by pale cyan. Methylation sites are circled in red, while pseudouridylation sites are indicated in blue. (**A**) Methylation and pseudouridylation sites are increased in BSF (+y). There seems to be a cluster of methylation sites in the SSU near the E-site. There is also a cluster of methylation sites near the nascent polypeptide exit tunnel as indicated by uL22 and near uL13 and uL3. uL13 has extra-ribosomal functions in mammals while uL3 has increased expression in certain mammalian tissues [17]. (**B**) Methylation and pseudouridylation sites in BSF (−y). Left figure shows a view of 180 degrees along the y-axis of (**A**); right side shows a view of 280 degrees along the y-axis of (**A**) and a magnification of 60 Å, highlighting helices composing the PTC. Pseudouridylation sites seem to cluster in the SSU, while methylation appears to cluster in the LSU on this side. (**C**) Increased methylation sites in PCF. Methylation sites appear more diffused in PCF parasites than in BSF forms. The helices composing the PTC are highlighted with the same color pattern as in (**B**). There are also more methylation sites in PCF than BSF.

Like *Plasmodium* spp., the main source of ribosome heterogeneity with potential functional specialization described in *Trypanosoma* spp. is the rRNA. For instance, it has been reported that pseudouridylation (Ψ) is developmentally regulated in two stages of *T. brucei*, where 21 positions on the rRNA were hyper-pseudouridylated in the bloodstream form (BSF) [106] (Figure 3A,B and Figure 4A,B). Interestingly, four domains located in the peptidyl-transferase center (PTC) were hypermodified (H69, H89, H90, and H92), where H69 and H89 have 4 hypermodified positions, while H90 and H92 have one each (Figure 4B). Additionally, overexpression of snoRNAs that guide the modifications of H69 accelerates the growth of polycyclic forms (PCF) at an elevated temperature (30 °C), which suggests that hyper-pseudouridylation participates in the ability of the parasite to maintain ribosome function despite the temperature changes between hosts [106] (Figure 3C).

Likewise, it has been described that several 2′-O methylations sites of rRNA are differentially regulated at the two stages of the life cycle of *T. brucei.* Specifically, the increased methylations observed in 18 and 36 positions in BSF and PCF, respectively (Figure 3A,B and Figure 4A–C); curiously, these modifications are close to functional domains of rRNA such as E, P, and A sites and PTC [107] (Figure 4B,C). Although the precise role of these rRNA modifications in each stage of *T. brucei* has not been discovered, their closeness to functional domains of the ribosome could improve the translation of specific subsets of mRNA and support the existence of different populations of ribosomes with specific functions during the *Trypanosoma* life cycle.

On the other hand, proteomic analyses of *T. cruzi* epimastigotes showed the overexpression of 33 ribosomal proteins (17 from the large subunit and 16 from the small subunit) in the exponential phase compared with stationary phase in vitro; in addition, four ribosomal proteins were uniquely expressed in the exponential phase (L7Ae/eL8, L27/eL27, L34/eL34, and one acidic RP) and one expressed exclusively in the stationary phase (S12), highlighting the importance of ribosomes in stages’ transitioning [108].

Another layer of translational control is achieved by ribosome-associated factors. In a recent study, the protein TbRACK1 was found in association with ribosomes and elongation factor 1A in *Trypanosoma brucei* and is important for the translational control of cytokinesis [109]. However, more studies are needed to understand what factors can bind ribosomes and what are their role in translational control.

### 3.2. Leishmania spp.

**Figure 5:**
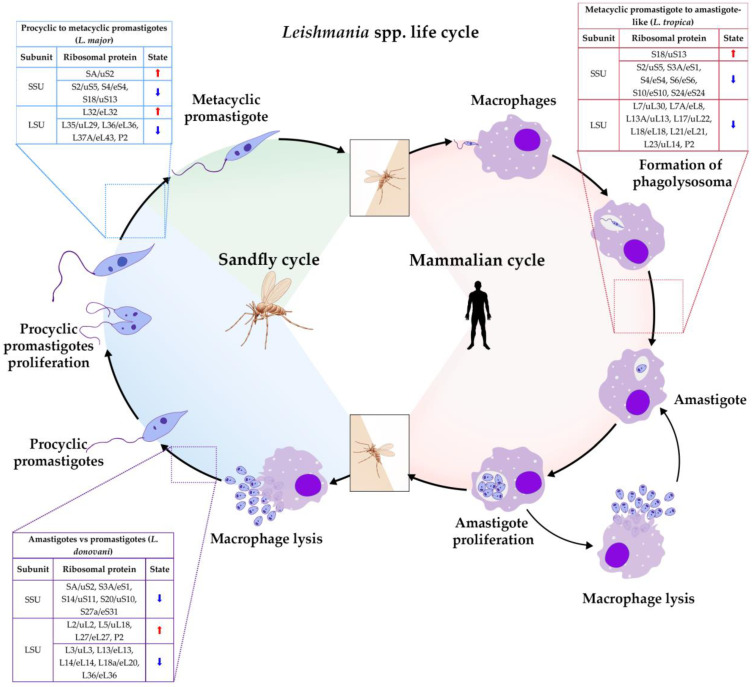
Ribosomal proteins (RP) developmentally regulated in *Leishmania*. Tables show up-regulated (⬆) and down-regulated (⬇) RPs from SSU and LSU. The blue Table illustrates RPs regulated in the differentiation of *L. major* from procyclic to metacyclic promastigotes [112]. The light red Table includes RPs regulated in the differentiation of *L. tropica* from metacyclic promastigotes to amastigotes-like [113]. The purple table shows RPs differentially expressed in amastigotes compared to promastigotes of *L. donovani* [114].

**Figure 6:**
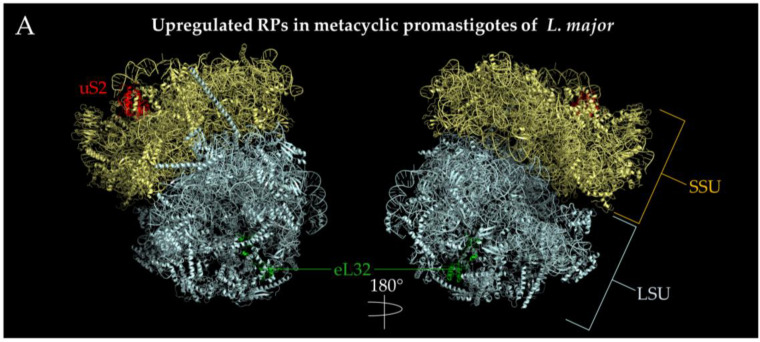

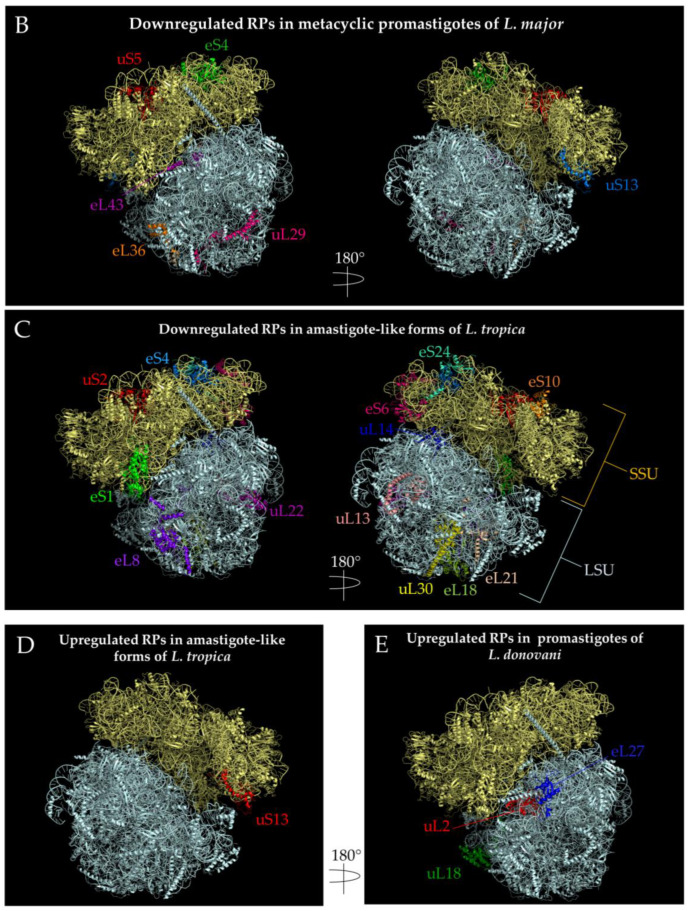

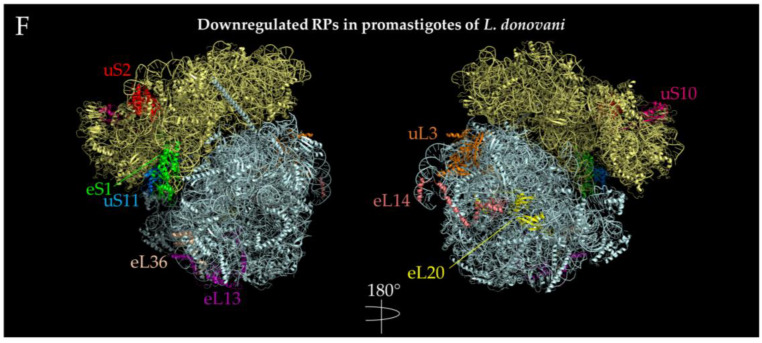
Localization of ribosomal proteins developmentally regulated in *Leishmania* spp. PDB 5T2A [121] ribosome structures were used to visualize the ribosomal proteins using PyMOL [110]. The SSU is indicated by pale yellow, while the LSU is indicated by pale cyan. Regulated proteins are highlighted and denoted next to the protein with their respective colors. (**A**) Upregulated ribosomal proteins in the transition from procyclic to metacyclic promastigotes in *Leishmania major*. LSUα, part of the 26S rRNA, was hidden to better illustrate the location of eL32. (**B**) Downregulated ribosomal proteins in the transition from procyclic to metacyclic promastigotes in *Leishmania major*. P2 is not pictured as PDB 5T2A does not contain a solved structure of this protein. (**C**). Downregulated ribosomal proteins in the transition from metacyclic promastigote to amastigote-like forms in *L. tropica*. Both the +y and -y directions for this stage of development were depicted. (**D**) Upregulated ribosomal proteins in the transition from metacyclic promastigotes to amastigote-like forms in *L. tropica*. The view of only one side of the ribosome is shown, which is 180 degrees from C. (**E**) Upregulated ribosomal proteins in the transition from amastigotes to promastigotes in *L. donovani*. The view of only one side of the ribosome is shown containing all three ribosomal proteins. P2 is not pictured as PDB 5T2A does not contain a solved structure of this protein. (**F**) Downregulated ribosomal proteins in the transition from amastigotes to promastigotes *L. donovani*. The +y and –y ribosome directions are indicated by a 180 degree turn. eS31 was not depicted since 5T2A does not have that coordinate.

Regarding *Leishmania* spp., there is limited information of rRNA or RP heterogeneity related to specialized ribosomes that are regulated during development; however, an interesting study about experimental evolution showed that *L. donovani* gains fitness at the cost of infectivity loss as an adaptation to the environment [111]. The authors, through genomic, transcriptomic, and proteomic analyses, demonstrated that some biological processes such as “ribosome biogenesis”, “ribosome assembly” and “rRNA processing” have an important role in fitness gain by forming “fitness-adapted ribosomes”, which can change the translation specificity or efficiency and allows the environment adaptation of *Leishmania* to changing conditions [111]. Likewise, a quantitative proteomic analysis also revealed that some ribosomal proteins are differentially expressed in the life cycle of *L. tropica*, *L. major*, and *L. donovani,* being up or down-regulated in different stages of the differentiation process in all species, indicating that ribosomal proteins are involved in the most important pathways of the metacyclogenesis mechanisms [112,113,114] (Figure 5 and Figure 6A–F).

All these characteristics observed in Trypanosomatids’ ribosomes have something in common: the involvement of small nucleolar RNAs (snoRNAs). snoRNAs are non-coding RNA present in nucleoli of eukaryotic cells which are mainly classified into two groups: C/D box and H/ACAbox snoRNAs, which participate in the processing of rRNA molecules by adding 2′-O-methylations and pseudouridylations (Ψ), respectively, although a third type of snoRNAs was recently described, the small Cajal body-specific RNAs (scaRNAs), which are in Cajal bodies and have both C/D and H/ACA structures [115,116,117]. They bind to partner proteins forming a ribonucleoprotein complex (RNP), which guides the recognition and tethering of target RNAs, thereby specifying the modification sites [115]. Unlike other eukaryotes, where around six snoRNAs are heading rRNA processing, Trypanosomatids have at least 18 snoRNAs having this function, which is the highest number reported [118,119]. Additionally, snoRNAs have shown differential developmental expression in *T. brucei* [106,107] and *L. donovani* [111], which, taking into account the importance of snoRNAs in ribosome assembly and biogenesis, could be related to the production of specialized ribosomes and contribute to the adaptation of these parasitic protozoa to host switching.

*Leishmania species* can exhibit the ribosome heterogeneity through ribosome-associated factors. LiAlba1 and LiAlba3 are RNA binding proteins controlling developmental gene expressions in *Leishmania infantum* [120]. These proteins can form complex interactions with ribosomal subunits, RNA binding proteins and translation factors. Their increased association with the ribosomal subunits is observed under conditions of decreased translation and support their role in translational repression. Interestingly, these proteins display a differential localization during parasite development.

### 3.3. Trypanosomatid Mitoribosomes

Trypanosomatid mitoribosomes have the most pronounced compositional and architectural deviations so far. They are larger and more complex than any other mitoribosome described to date, with a molecular weight of 4.5 MDa, highly reduced rRNA content (1796 nt) located in the core of the ribosome and an expanded repertoire of proteins (127) acting as a shell for rRNA [63]. Roughly half of the ribosomal proteins found in trypanosomal mitochondria are unique to this species [122]. The RNA/protein ratio of *T. brucei* is 1:6, differing from the 2:1 ratio found in *E. coli*, 1:1 ratio of *S. cerevisiae* mitochondria, and the 1:2 ratio found in mammalian mitochondria (Table 1) [63,123,124].

These mitoribosomes are also characterized by a prominent SSU, which is even bigger than LSU [63], as well as the presence of unusual 45S SSU* complexes composed of 9S rRNA, at least 39 ribosomal proteins, pentatricopeptides repeats (PPR) and other proteins not typically found in ribosomes, such as rhodanese-like protein (Rhod) [66,125,126]. This complex has shown an important role in the growth of procyclic forms as well as the synthesis of COI and Cyb in *T. brucei* and consequently, affecting the production of ATP [126]. Likewise, mitoribosomal protein MRPS12/uS12m is the only ribosomal protein encoded by kinetoplast DNA, which undergoes an extensive U-insertion/deletion editing. It has observed that MRPS12/uS12m mRNA editing is essential for mitochondrial translation, since perturbations of the editing system results in an inhibition of the mitochondrial protein synthesis, affecting the production of Cyb and COI, with a parallel reduction in 45S SSU* complexes [127]. Both studies highlight that such regulation becomes necessary during the differentiation of bloodstream forms (BSF) into the insect procyclic forms (PCF) of *T. brucei*. It must be accompanied by a transition from mitochondrion lacking cytochrome-mediated electron transport chain into a fully functional system of oxidative phosphorylation [126,127], since in BSF the ATP is produced via substrate level phosphorylation and is hydrolyzed by the F_1_F_0_ ATP synthase to generate mitochondrial trans-membrane potential [128,129].

## 4. Role of Ribosome Specialization in Parasitism

Protozoa parasites have complex life cycles, switching between two hosts or between environment-host, which has turned them into unique organisms with elaborate and complex machinery that allows their adaptation to adverse environments [130]. At the molecular level, it has been observed that protozoa parasites can exhibit changes in the genome [131,132,133], transcriptome [134,135,136], translatome [137], and proteome [138,139,140] when facing stressful factors and in different developmental stages. In this way, the regulation of mRNA translation becomes essential for parasite invasion, proliferation, and its response to changes in temperature, nutrient depletion, and the immune response of the host.

Several proteomic analyses have shown that ribosomal proteins as well as proteins related to ribosome biogenesis are some of the most up or downregulated proteins in the transition stage [108,112,113,141]. However, it is necessary to highlight that some ribosomal proteins exhibit extraribosomal functions in other organisms, such as acting as DNA endonucleases (RPS3/uS3) [142,143], helping in antiviral responses (RPL10/uL16, RPL13A/uL13) [144,145], and even being part of the small nucleolar ribonucleoproteins (snoRNP) (RPL7/uL30) [146]; some act in autoregulation of RP synthesis and as sentinels in ribosome biosynthesis, among other functions [147,148,149,150]. Therefore, currently, it remains unknown if the ribosomal proteins differentially expressed in stage transition have extraribosomal functions or may be associated with the formation of specialized ribosomes in protozoa parasites.

In addition to ribosomal proteins, other molecules such as rRNA, snoRNAs, ribosome-associated proteins and non-coding RNA play fundamental roles in the biogenesis of ribosomes, favoring base modification, protein binding, bond establishment, sequence cleavage, and recently have been recognized as regulators of ribosome functions and translation [109,111,118,120,151,152,153,154,155,156,157,158,159,160,161]. Non-coding RNAs (ncRNAs) are present in all three domains of life, and some of them have shown a special stress-specific expression, inhibiting protein production on the global scale or having a stimulating effect on translation by interacting with translating ribosomes in a stress-dependent manner [157,158]. In protozoa parasites, it has been also described that certain ncRNAs are developmentally regulated and/or respond to stress, they can be associated with ribosomes and have a potential role in translation regulation in *T. brucei* and *L. infantum* [154,159,160]. Furthermore, long ncRNAs that promote the parasite differentiation in *T. brucei* have also been reported [161]. All these molecules could have an important role in developing specialized ribosomes that regulate translation during the hosts switching, or during its adaption to different challenges such as starvation, heat-shock, and oxidative stress during infection [130].

A recent study revealed that infection with *Toxoplasma gondii* leads to a dramatic change in the ribosome composition in mammalian macrophages and selective reprogramming of host mRNA translation [162]. Parasite infection induces upregulation of many ribosomal proteins in the host and activates mTORC1. The inhibition of host mTORC1 leads to the reduction in parasite replication, supporting that manipulation of host ribosome composition and selective reprogramming of host translatome are important strategies for parasite survival during infection.

The functional specialization of ribosomes is poorly understood in protozoa parasites; studies carried out on other model organisms have shown interesting mechanisms of ribosome specialization as a response to stress. In *E. coli,* stressful conditions such as the presence of antibiotics triggers the stress-induced toxin-antitoxin (TA) system *masEF,* which encodes the labile antitoxin MazE and the stable toxin MazF. However, due to the short lifespan of MazE, it is quickly degraded, and MazF exerts its toxic effects. MazF is an endonuclease that cleaves single stranded mRNA at ACA sequences, nevertheless it also cleaves the 16S rRNA within 30S ribosomal subunit in a region required for translation initiation of canonical mRNAs (anti-Shine-Dalgarno region, aSD). The subpopulation of ribosomes lacking the 43 nucleotides from 3’ terminus of 16S rRNA selectively translate leaderless mRNA in vivo and in vitro, as a possible response to stress adaptation of *E. coli* [163]. Likewise, when *E. coli* faces nutrient limitations, it has observed an upregulation of the operon *rrnH*, which encodes the 16S rRNA gene *rrsH.* The presence of rrsH-bearing ribosomes leads to changes in the expression of stress response genes regulated by the RpoS sigma factor, and alters phenotypic traits such as antibiotic sensitivity, biofilm formation, and cell motility [164]. On the other hand, *Haloarcula marismortui* is a halophilic archaeon which contains three rRNA operons (*rrnA*, *rrnB*, and *rrnC*), operons A and C are identical, but operon B has a highly divergent nucleotide sequence. It has been observed that at high temperatures (50 °C) operon B displays four times higher expression levels than operons A and C together; likewise, strain lacking operon B grew slower at high temperatures [165], which highlights the importance of specialized paralogues in archaeon survival at different temperatures.

Thus, it is logical to conclude that heterogeneities in ribosomes could lead to a functional specialization directed to produce proteins with important roles for parasite survival; however, more deep studies are required to determine the functional consequences of ribosomal specialization in protozoa parasites. Specialized ribosome components could also be used as new pharmaceutical targets or as antigens in the development of vaccines [166,167,168].

## 5. Conclusions

Recent evidence indicates that specialized ribosomes can support selective translation of a subset of mRNAs, which makes ribosomes an important regulator of translational control. Our current knowledge about ribosome specialization in protozoa parasites remains very limited. Most of the ribosome heterogeneities reported here are caused by modifications of rRNA, changes in DNA coding rRNAs and heterogeneity in ribosomal proteins. Despite multiple proteomic studies showing RPs exclusively expressed in different developmental stages, more studies are required to assess the role of ribosomal proteins in the formation of specialized ribosomes and the biological consequences of such specializations. The functional role of rRNA modifications in the life cycle of the parasites also remains obscure. Notwithstanding, studies about ribosome specialization in parasitic protozoa are scarce, some protozoa such as Trypanosomatids can serve as an excellent model organisms to study ribosome specialization since they have a limited transcriptional control, while regulation of translation is the major route to control gene expression [169,170]. On the other hand, techniques such as cryoEM have made it possible to deeply unravel the structures of ribosomes at resolutions never seen before. More studies of this type are required to know in depth about the variations in the structure of protozoa parasites’ ribosomes and how these differences provide a different landscape and contribute to selective translation. The functional role that specialized ribosomes and mitoribosomes play in the response of the parasite to environmental conditions, the life cycle of the parasite, or its virulence is one of the major unanswered fundamental questions that require further studies.

## Figures and Tables

**Table 1:** Composition of cytosolic and mitochondrial ribosomes in different organisms.

	*E. coli* Ribosome [61]	*S. cerevisiae* Mitoribosome [62]	*T. brucei* Mitoribosome [63]	*H. sapiens* Mitoribosome [64]	Eukaryotic Cytosol Ribosomes [61]
**Ribosome**					
Sedimentation coefficient	70S	74S	~60S [65]	55S	80S
Molecular weight	2.3 MDa	3–3.3 MDa	4.5 MDa	2.7 MDa	3.3–4.3 MDa
Number of rRNAs	3	2	2	3	4
Number of proteins	55	80	127	83	79–80
**Large subunit**					
Sedimentation coefficient	50S	54S	40S *	39S	60S
Number of rRNAs	23S (2904 nt)	21S (3296 nt)	12S (1176 nt)	16S (1569 nt)	26S–28S (3396–5034 nt)
					5.8S (156–158 nt)
	5S (120 nt)			tRNA (73–75 nt)	5S (120–121 nt)
Number of proteins	33	46	72	50	46–47
**Small subunit**					
Sedimentation coefficient	30S	37S	30S *	28S	40S
Number of rRNAs	16S (1542 nt)	15S (1649 nt)	9S (620 nt)	12S (962 nt)	18S (1800–1870 nt)
Number of proteins	22	34	55	30	33

* Sedimentation coefficient belongs to *Leishmania tarentolae* [66].

## Data Availability

Not applicable.

## Abbreviations

aSD	Anti-Shine-Dalgarno region
BSF	Bloodstream forms
CryoEM	Cryo-Electron Microscopy
COI	Cytochrome *c* oxidase subunit I
Cyb	Cytochrome *b*
eIF4E	Eukaryotic initiation factor 4E
FMRP	Fragile X Mental Retardation Protein
H69	Helix 69
LSU	Large subunit
LiAlba1	*Leishmania infantum* Acetylation lowers binding affinity protein 1
LiAlba3	*Leishmania infantum* Acetylation lowers binding affinity protein 3
MDa	Mega Dalton
mESCs	Mouse embryonic stem cells
mRNA	Messenger RNA
MRP	Mitochondrial ribosomal protein
mtDNA	Mitochondrial DNA
mtETC	Mitochondrial electron transport chain
mTORC1	Mammalian target of rapamycin complex 1
ncRNA	Non-coding RNA
OOD	Oocyst development defect
PCF	Polycyclic forms
PDB	Protein database
PPR	Pentatricopeptides repeats
PTC	Peptidyl transferase center
RACK1	Receptor for activated C kinase 1
rancRNA	Ribosome associated non-coding RNA
rDNA	Ribosomal DNA
RNP	Ribonucleoprotein
RP	Ribosomal protein
RPL	Ribosomal protein L
RPS	Ribosomal protein S
rRNA	Ribosomal RNA
SAR	Social amoebae ribosomes
scaRNAs	Small Cajal body-specific RNAs
snoRNA	Small nucleolar RNA
SSU	Small subunit
TA	Toxin-Antitoxin
TbRACK1	*Trypanosoma brucei* Receptor of activated protein C kinase 1
VAR	Vegetative amoebae ribosomes
Ψ	Pseudouridylation

## References

[1] Melnikov S., Ben-Shem A., Garreau de Loubresse N., Jenner L., Yusupova G., Yusupov M. (2012). One core, two shells: Bacterial and eukaryotic ribosomes. Nat. Struct. Mol. Biol..

[2] Ben-Shem A., Garreau de Loubresse N., Melnikov S., Jenner L., Yusupova G., Yusupov M. (2011). The structure of the eukaryotic ribosome at 3.0 A resolution. Science.

[3] Field K.G., Olsen G.J., Lane D.J., Giovannoni S.J., Ghiselin M.T., Raff E.C., Pace N.R., Raff R.A. (1988). Molecular phylogeny of the animal kingdom. Science.

[4] Xue S., Barna M. (2012). Specialized ribosomes: A new frontier in gene regulation and organismal biology. Nat. Rev. Mol. Cell Biol..

[5] Van Duin J., Kurland C.G. (1970). Functional heterogeneity of the 30S ribosomal subunit of *E. coli*. Mol. Gen. Genet..

[6] Deusser E. (1972). Heterogeneity of ribosomal populations in *Escherichia coli* cells grown in different media. Mol. Gen. Genet..

[7] Leipold R.J., Dhurjati P. (1993). Specialized ribosomes in *Escherichia coli*. Biotechnol. Prog..

[8] Ramagopal S. (1992). Are eukaryotic ribosomes heterogeneous? Affirmations on the horizon. Biochem. Cell Biol..

[9] Hui A., de Boer H.A. (1987). Specialized ribosome system: Preferential translation of a single mRNA species by a subpopulation of mutated ribosomes in *Escherichia coli*. Proc. Natl. Acad. Sci. USA.

[10] Mauro V.P., Edelman G.M. (2002). The ribosome filter hypothesis. Proc. Natl. Acad. Sci. USA.

[11] Mauro V.P., Edelman G.M. (2007). The ribosome filter redux. Cell Cycle.

[12] Shi Z., Fujii K., Kovary K.M., Genuth N.R., Rost H.L., Teruel M.N., Barna M. (2017). Heterogeneous Ribosomes Preferentially Translate Distinct Subpools of mRNAs Genome-wide. Mol. Cell.

[13] Bauer J.W., Brandl C., Haubenreisser O., Wimmer B., Weber M., Karl T., Klausegger A., Breitenbach M., Hintner H., von der Haar T. (2013). Specialized yeast ribosomes: A customized tool for selective mRNA translation. PLoS ONE.

[14] Xue S., Tian S., Fujii K., Kladwang W., Das R., Barna M. (2015). RNA regulons in Hox 5′ UTRs confer ribosome specificity to gene regulation. Nature.

[15] Simsek D., Tiu G.C., Flynn R.A., Byeon G.W., Leppek K., Xu A.F., Chang H.Y., Barna M. (2017). The Mammalian Ribo-interactome Reveals Ribosome Functional Diversity and Heterogeneity. Cell.

[16] Ferretti M.B., Ghalei H., Ward E.A., Potts E.L., Karbstein K. (2017). Rps26 directs mRNA-specific translation by recognition of Kozak sequence elements. Nat. Struct. Mol. Biol..

[17] Miller S.C., MacDonald C.C., Kellogg M.K., Karamysheva Z.N., Karamyshev A.L. (2023). Specialized Ribosomes in Health and Disease. Int. J. Mol. Sci..

[18] Genuth N.R., Shi Z., Kunimoto K., Hung V., Xu A.F., Kerr C.H., Tiu G.C., Oses-Prieto J.A., Salomon-Shulman R.E.A., Axelrod J.D. (2022). A stem cell roadmap of ribosome heterogeneity reveals a function for RPL10A in mesoderm production. Nat. Commun..

[19] Kondrashov N., Pusic A., Stumpf C.R., Shimizu K., Hsieh A.C., Ishijima J., Shiroishi T., Barna M. (2011). Ribosome-mediated specificity in Hox mRNA translation and vertebrate tissue patterning. Cell.

[20] Sugihara Y., Honda H., Iida T., Morinaga T., Hino S., Okajima T., Matsuda T., Nadano D. (2010). Proteomic analysis of rodent ribosomes revealed heterogeneity including ribosomal proteins L10-like, L22-like 1, and L39-like. J. Proteome Res..

[21] Sugihara Y., Sadohara E., Yonezawa K., Kugo M., Oshima K., Matsuda T., Nadano D. (2013). Identification and expression of an autosomal paralogue of ribosomal protein S4, X-linked, in mice: Potential involvement of testis-specific ribosomal proteins in translation and spermatogenesis. Gene.

[22] Lopes A.M., Miguel R.N., Sargent C.A., Ellis P.J., Amorim A., Affara N.A. (2010). The human RPS4 paralogue on Yq11.223 encodes a structurally conserved ribosomal protein and is preferentially expressed during spermatogenesis. BMC Mol. Biol..

[23] Zou Q., Yang L., Shi R., Qi Y., Zhang X., Qi H. (2021). Proteostasis regulated by testis-specific ribosomal protein RPL39L maintains mouse spermatogenesis. iScience.

[24] Fang Y., Zong Q., He Z., Liu C., Wang Y.F. (2021). Knockdown of RpL36 in testes impairs spermatogenesis in *Drosophila melanogaster*. J. Exp. Zool. Part B Mol. Dev. Evol..

[25] Mageeney C.M., Ware V.C. (2019). Specialized eRpL22 paralogue-specific ribosomes regulate specific mRNA translation in spermatogenesis in *Drosophila melanogaster*. Mol. Biol. Cell.

[26] Li H., Huo Y., He X., Yao L., Zhang H., Cui Y., Xiao H., Xie W., Zhang D., Wang Y. (2022). A male germ-cell-specific ribosome controls male fertility. Nature.

[27] Ghulam M.M., Catala M., Abou Elela S. (2020). Differential expression of duplicated ribosomal protein genes modifies ribosome composition in response to stress. Nucleic Acids Res..

[28] Ghulam M.M., Catala M., Reulet G., Scott M.S., Abou Elela S. (2022). Duplicated ribosomal protein paralogs promote alternative translation and drug resistance. Nat. Commun..

[29] Guo H. (2018). Specialized ribosomes and the control of translation. Biochem. Soc. Trans..

[30] Filipovska A., Rackham O. (2013). Specialization from synthesis: How ribosome diversity can customize protein function. FEBS Lett..

[31] Sauert M., Temmel H., Moll I. (2015). Heterogeneity of the translational machinery: Variations on a common theme. Biochimie.

[32] Shi Z., Barna M. (2015). Translating the genome in time and space: Specialized ribosomes, RNA regulons, and RNA-binding proteins. Annu. Rev. Cell Dev. Biol..

[33] Genuth N.R., Barna M. (2018). Heterogeneity and specialized functions of translation machinery: From genes to organisms. Nat. Rev. Genet..

[34] Li D., Wang J. (2020). Ribosome heterogeneity in stem cells and development. J. Cell Biol..

[35] Gay D.M., Lund A.H., Jansson M.D. (2022). Translational control through ribosome heterogeneity and functional specialization. Trends Biochem. Sci..

[36] Joo M., Yeom J.H., Choi Y., Jun H., Song W., Kim H.L., Lee K., Shin E. (2022). Specialised ribosomes as versatile regulators of gene expression. RNA Biol..

[37] Komili S., Farny N.G., Roth F.P., Silver P.A. (2007). Functional specificity among ribosomal proteins regulates gene expression. Cell.

[38] Imami K., Milek M., Bogdanow B., Yasuda T., Kastelic N., Zauber H., Ishihama Y., Landthaler M., Selbach M. (2018). Phosphorylation of the Ribosomal Protein RPL12/uL11 Affects Translation during Mitosis. Mol. Cell.

[39] Ladror D.T., Frey B.L., Scalf M., Levenstein M.E., Artymiuk J.M., Smith L.M. (2014). Methylation of yeast ribosomal protein S2 is elevated during stationary phase growth conditions. Biochem. Biophys. Res. Commun..

[40] Parks M.M., Kurylo C.M., Dass R.A., Bojmar L., Lyden D., Vincent C.T., Blanchard S.C. (2018). Variant ribosomal RNA alleles are conserved and exhibit tissue-specific expression. Sci. Adv..

[41] Decatur W.A., Fournier M.J. (2002). rRNA modifications and ribosome function. Trends Biochem. Sci..

[42] Charette M., Gray M.W. (2000). Pseudouridine in RNA: What, where, how, and why. IUBMB Life.

[43] Ofengand J., Fournier M.J. (2014). The Pseudouridine Residues of rRNA: Number, Location, Biosynthesis, and Function. Modif. Ed. RNA.

[44] Maden B.E. (1990). The numerous modified nucleotides in eukaryotic ribosomal RNA. Prog. Nucleic Acid Res. Mol. Biol..

[45] Lane B.G., Ofengand J., Gray M.W. (1995). Pseudouridine and O2′-methylated nucleosides. Significance of their selective occurrence in rRNA domains that function in ribosome-catalyzed synthesis of the peptide bonds in proteins. Biochimie.

[46] Smith J.E., Cooperman B.S., Mitchell P. (1992). Methylation sites in *Escherichia coli* ribosomal RNA: Localization and identification of four new sites of methylation in 23S rRNA. Biochemistry.

[47] Jack K., Bellodi C., Landry D.M., Niederer R.O., Meskauskas A., Musalgaonkar S., Kopmar N., Krasnykh O., Dean A.M., Thompson S.R. (2011). rRNA pseudouridylation defects affect ribosomal ligand binding and translational fidelity from yeast to human cells. Mol. Cell.

[48] Sloan K.E., Warda A.S., Sharma S., Entian K.D., Lafontaine D.L.J., Bohnsack M.T. (2017). Tuning the ribosome: The influence of rRNA modification on eukaryotic ribosome biogenesis and function. RNA Biol..

[49] Adams D.R., Ron D., Kiely P.A. (2011). RACK1, A multifaceted scaffolding protein: Structure and function. Cell Commun. Signal..

[50] Gallo S., Ricciardi S., Manfrini N., Pesce E., Oliveto S., Calamita P., Mancino M., Maffioli E., Moro M., Crosti M. (2018). RACK1 Specifically Regulates Translation through Its Binding to Ribosomes. Mol. Cell. Biol..

[51] Darnell J.C., Van Driesche S.J., Zhang C., Hung K.Y., Mele A., Fraser C.E., Stone E.F., Chen C., Fak J.J., Chi S.W. (2011). FMRP stalls ribosomal translocation on mRNAs linked to synaptic function and autism. Cell.

[52] O’Brien T.W. (2002). Evolution of a protein-rich mitochondrial ribosome: Implications for human genetic disease. Gene.

[53] Sharma M.R., Koc E.C., Datta P.P., Booth T.M., Spremulli L.L., Agrawal R.K. (2003). Structure of the mammalian mitochondrial ribosome reveals an expanded functional role for its component proteins. Cell.

[54] O’Brien T.W. (2003). Properties of human mitochondrial ribosomes. IUBMB Life.

[55] Koc E.C., Burkhart W., Blackburn K., Moseley A., Spremulli L.L. (2001). The small subunit of the mammalian mitochondrial ribosome. Identification of the full complement of ribosomal proteins present. J. Biol. Chem..

[56] Koc E.C., Burkhart W., Blackburn K., Moyer M.B., Schlatzer D.M., Moseley A., Spremulli L.L. (2001). The large subunit of the mammalian mitochondrial ribosome. Analysis of the complement of ribosomal proteins present. J. Biol. Chem..

[57] Koc E.C., Cimen H., Kumcuoglu B., Abu N., Akpinar G., Haque M.E., Spremulli L.L., Koc H. (2013). Identification and characterization of CHCHD1, AURKAIP1, and CRIF1 as new members of the mammalian mitochondrial ribosome. Front. Physiol..

[58] Suzuki T., Terasaki M., Takemoto-Hori C., Hanada T., Ueda T., Wada A., Watanabe K. (2001). Structural compensation for the deficit of rRNA with proteins in the mammalian mitochondrial ribosome. Systematic analysis of protein components of the large ribosomal subunit from mammalian mitochondria. J. Biol. Chem..

[59] Suzuki T., Terasaki M., Takemoto-Hori C., Hanada T., Ueda T., Wada A., Watanabe K. (2001). Proteomic analysis of the mammalian mitochondrial ribosome. Identification of protein components in the 28 S small subunit. J. Biol. Chem..

[60] Ott M., Herrmann J.M. (2010). Co-translational membrane insertion of mitochondrially encoded proteins. Biochim. Biophys. Acta.

[61] Yusupova G., Yusupov M. (2014). High-resolution structure of the eukaryotic 80S ribosome. Annu. Rev. Biochem..

[62] Desai N., Brown A., Amunts A., Ramakrishnan V. (2017). The structure of the yeast mitochondrial ribosome. Science.

[63] Ramrath D.J.F., Niemann M., Leibundgut M., Bieri P., Prange C., Horn E.K., Leitner A., Boehringer D., Schneider A., Ban N. (2018). Evolutionary shift toward protein-based architecture in trypanosomal mitochondrial ribosomes. Science.

[64] Amunts A., Brown A., Toots J., Scheres S.H.W., Ramakrishnan V. (2015). Ribosome. The structure of the human mitochondrial ribosome. Science.

[65] Zikova A., Panigrahi A.K., Dalley R.A., Acestor N., Anupama A., Ogata Y., Myler P.J., Stuart K. (2008). *Trypanosoma brucei* mitochondrial ribosomes: Affinity purification and component identification by mass spectrometry. Mol. Cell. Proteom..

[66] Maslov D.A., Sharma M.R., Butler E., Falick A.M., Gingery M., Agrawal R.K., Spremulli L.L., Simpson L. (2006). Isolation and characterization of mitochondrial ribosomes and ribosomal subunits from *Leishmania tarentolae*. Mol. Biochem. Parasitol..

[67] Kessin R.H. (2010). Dictyostelium: Evolution, Cell Biology, and the Development of Multicellularity.

[68] Dunn J.D., Bosmani C., Barisch C., Raykov L., Lefrancois L.H., Cardenal-Munoz E., Lopez-Jimenez A.T., Soldati T. (2017). Eat Prey, Live: *Dictyostelium discoideum* As a Model for Cell-Autonomous Defenses. Front. Immunol..

[69] Ramagopal S., Ennis H.L. (1981). Regulation of synthesis of cell-specific ribosomal proteins during differentiation of *Dictyostelium discoideum*. Proc. Natl. Acad. Sci. USA.

[70] Ramagopal S. (1990). Induction of cell-specific ribosomal proteins in aggregation-competent nonmorphogenetic *Dictyostelium discoideum*. Biochem. Cell Biol..

[71] Ramagopal S. (1991). Covalent modifications of ribosomal proteins in growing and aggregation-competent *Dictyostelium discoideum*: Phosphorylation and methylation. Biochem. Cell Biol..

[72] Ramagopal S. (1992). The *Dictyostelium ribosome*: Biochemistry, molecular biology, and developmental regulation. Biochem. Cell Biol..

[73] Ban N., Beckmann R., Cate J.H., Dinman J.D., Dragon F., Ellis S.R., Lafontaine D.L., Lindahl L., Liljas A., Lipton J.M. (2014). A new system for naming ribosomal proteins. Curr. Opin. Struct. Biol..

[74] Moore J. (1991). Studies of the Relationship between mRNA Stability and Gene Function in Saccharomyces cerevisiae.

[75] Vembar S.S., Droll D., Scherf A. (2016). Translational regulation in blood stages of the malaria parasite *Plasmodium* spp.: Systems-wide studies pave the way. Wiley Interdiscip. Rev. RNA.

[76] McCutchan T.F., Li J., McConkey G.A., Rogers M.J., Waters A.P. (1995). The cytoplasmic ribosomal RNAs of *Plasmodium* spp.. Parasitol. Today.

[77] Gunderson J.H., Sogin M.L., Wollett G., Hollingdale M., de la Cruz V.F., Waters A.P., McCutchan T.F. (1987). Structurally distinct, stage-specific ribosomes occur in *Plasmodium*. Science.

[78] Waters A.P., Syin C., McCutchan T.F. (1989). Developmental regulation of stage-specific ribosome populations in *Plasmodium*. Nature.

[79] Li J., McConkey G.A., Rogers M.J., Waters A.P., McCutchan T.R. (1994). *Plasmodium*: The developmentally regulated ribosome. Exp. Parasitol..

[80] Waters A.P., van Spaendonk R.M., Ramesar J., Vervenne R.A., Dirks R.W., Thompson J., Janse C.J. (1997). Species-specific regulation and switching of transcription between stage-specific ribosomal RNA genes in *Plasmodium berghei*. J. Biol. Chem..

[81] Thompson J., van Spaendonk R.M., Choudhuri R., Sinden R.E., Janse C.J., Waters A.P. (1999). Heterogeneous ribosome populations are present in *Plasmodium berghei* during development in its vector. Mol. Microbiol..

[82] Velichutina I.V., Rogers M.J., McCutchan T.F., Liebman S.W. (1998). Chimeric rRNAs containing the GTPase centers of the developmentally regulated ribosomal rRNAs of *Plasmodium falciparum* are functionally distinct. RNA.

[83] Qi Y., Zhu F., Eastman R.T., Fu Y., Zilversmit M., Pattaradilokrat S., Hong L., Liu S., McCutchan T.F., Pan W. (2015). Regulation of *Plasmodium yoelii* oocyst development by strain- and stage-specific small-subunit rRNA. mBio.

[84] van Spaendonk R.M., Ramesar J., van Wigcheren A., Eling W., Beetsma A.L., van Gemert G.J., Hooghof J., Janse C.J., Waters A.P. (2001). Functional equivalence of structurally distinct ribosomes in the malaria parasite, *Plasmodium berghei*. J. Biol. Chem..

[85] Carraway M., Tzipori S., Widmer G. (1996). Identification of genetic heterogeneity in the *Cryptosporidium parvum* ribosomal repeat. Appl. Environ. Microbiol..

[86] Le Blancq S.M., Khramtsov N.V., Zamani F., Upton S.J., Wu T.W. (1997). Ribosomal RNA gene organization in *Cryptosporidium parvum*. Mol. Biochem. Parasitol..

[87] Erath J., Djuranovic S. (2022). Association of the receptor for activated C-kinase 1 with ribosomes in *Plasmodium falciparum*. J. Biol. Chem..

[88] Vaidya A.B., Mather M.W. (2009). Mitochondrial evolution and functions in malaria parasites. Annu. Rev. Microbiol..

[89] Evers F., Cabrera-Orefice A., Elurbe D.M., Kea-Te Lindert M., Boltryk S.D., Voss T.S., Huynen M.A., Brandt U., Kooij T.W.A. (2021). Composition and stage dynamics of mitochondrial complexes in *Plasmodium falciparum*. Nat. Commun..

[90] Feagin J.E., Harrell M.I., Lee J.C., Coe K.J., Sands B.H., Cannone J.J., Tami G., Schnare M.N., Gutell R.R. (2012). The fragmented mitochondrial ribosomal RNAs of *Plasmodium falciparum*. PLoS ONE.

[91] Dass S., Mather M.W., Morrisey J.M., Ling L., Vaidya A.B., Ke H. (2022). Transcriptional changes in *Plasmodium falciparum* upon conditional knock down of mitochondrial ribosomal proteins RSM22 and L23. PLoS ONE.

[92] Gupta A., Shah P., Haider A., Gupta K., Siddiqi M.I., Ralph S.A., Habib S. (2014). Reduced ribosomes of the apicoplast and mitochondrion of *Plasmodium* spp. and predicted interactions with antibiotics. Open Biol..

[93] Wilson R.J., Denny P.W., Preiser P.R., Rangachari K., Roberts K., Roy A., Whyte A., Strath M., Moore D.J., Moore P.W. (1996). Complete gene map of the plastid-like DNA of the malaria parasite *Plasmodium falciparum*. J. Mol. Biol..

[94] Arisue N., Hashimoto T., Mitsui H., Palacpac N.M., Kaneko A., Kawai S., Hasegawa M., Tanabe K., Horii T. (2012). The *Plasmodium* apicoplast genome: Conserved structure and close relationship of *P. ovale* to rodent malaria parasites. Mol. Biol. Evol..

[95] Ke H., Dass S., Morrisey J.M., Mather M.W., Vaidya A.B. (2018). The mitochondrial ribosomal protein L13 is critical for the structural and functional integrity of the mitochondrion in *Plasmodium falciparum*. J. Biol. Chem..

[96] Ling L., Mulaka M., Munro J., Dass S., Mather M.W., Riscoe M.K., Llinas M., Zhou J., Ke H. (2020). Genetic ablation of the mitoribosome in the malaria parasite *Plasmodium falciparum* sensitizes it to antimalarials that target mitochondrial functions. J. Biol. Chem..

[97] Kampoun T., Srichairatanakool S., Prommana P., Shaw P.J., Green J.L., Knuepfer E., Holder A.A., Uthaipibull C. (2022). Apicoplast ribosomal protein S10-V127M enhances artemisinin resistance of a Kelch13 transgenic *Plasmodium falciparum*. Malar. J..

[98] Zhang M., Wang C., Oberstaller J., Thomas P., Otto T.D., Casandra D., Boyapalle S., Adapa S.R., Xu S., Button-Simons K. (2021). The apicoplast link to fever-survival and artemisinin-resistance in the malaria parasite. Nat. Commun..

[99] Simpson L. (1986). Kinetoplast DNA in trypanosomid flagellates. Int. Rev. Cytol..

[100] Walder J.A., Eder P.S., Engman D.M., Brentano S.T., Walder R.Y., Knutzon D.S., Dorfman D.M., Donelson J.E. (1986). The 35-nucleotide spliced leader sequence is common to all trypanosome messenger RNA’s. Science.

[101] Lewdorowicz M., Yoffe Y., Zuberek J., Jemielity J., Stepinski J., Kierzek R., Stolarski R., Shapira M., Darzynkiewicz E. (2004). Chemical synthesis and binding activity of the trypanosomatid cap-4 structure. RNA.

[102] White T.C., Rudenko G., Borst P. (1986). Three small RNAs within the 10 kb trypanosome rRNA transcription unit are analogous to domain VII of other eukaryotic 28S rRNAs. Nucleic Acids Res..

[103] Campbell D.A., Kubo K., Clark C.G., Boothroyd J.C. (1987). Precise identification of cleavage sites involved in the unusual processing of trypanosome ribosomal RNA. J. Mol. Biol..

[104] Hashem Y., des Georges A., Fu J., Buss S.N., Jossinet F., Jobe A., Zhang Q., Liao H.Y., Grassucci R.A., Bajaj C. (2013). High-resolution cryo-electron microscopy structure of the *Trypanosoma brucei* ribosome. Nature.

[105] Liu Z., Gutierrez-Vargas C., Wei J., Grassucci R.A., Ramesh M., Espina N., Sun M., Tutuncuoglu B., Madison-Antenucci S., Woolford J.L. (2016). Structure and assembly model for the *Trypanosoma cruzi* 60S ribosomal subunit. Proc. Natl. Acad. Sci. USA.

[106] Chikne V., Doniger T., Rajan K.S., Bartok O., Eliaz D., Cohen-Chalamish S., Tschudi C., Unger R., Hashem Y., Kadener S. (2016). A pseudouridylation switch in rRNA is implicated in ribosome function during the life cycle of *Trypanosoma brucei*. Sci. Rep..

[107] Rajan K.S., Zhu Y., Adler K., Doniger T., Cohen-Chalamish S., Srivastava A., Shalev-Benami M., Matzov D., Unger R., Tschudi C. (2020). The large repertoire of 2′-O-methylation guided by C/D snoRNAs on *Trypanosoma brucei* rRNA. RNA Biol..

[108] Avila C.C., Mule S.N., Rosa-Fernandes L., Viner R., Barison M.J., Costa-Martins A.G., Oliveira G.S., Teixeira M.M.G., Marinho C.R.F., Silber A.M. (2018). Proteome-Wide Analysis of *Trypanosoma cruzi* Exponential and Stationary Growth Phases Reveals a Subcellular Compartment-Specific Regulation. Genes.

[109] Regmi S., Rothberg K.G., Hubbard J.G., Ruben L. (2008). The RACK1 signal anchor protein from Trypanosoma brucei associates with eukaryotic elongation factor 1A: A role for translational control in cytokinesis. Mol. Microbiol..

[110] (2022). The PyMOL Molecular Graphics System.

[111] Piel L., Rajan K.S., Bussotti G., Varet H., Legendre R., Proux C., Douche T., Giai-Gianetto Q., Chaze T., Cokelaer T. (2022). Experimental evolution links post-transcriptional regulation to *Leishmania* fitness gain. PLoS Pathog..

[112] Amiri-Dashatan N., Rezaei-Tavirani M., Zali H., Koushki M., Ahmadi N. (2020). Quantitative proteomic analysis reveals differentially expressed proteins in *Leishmania major* metacyclogenesis. Microb. Pathog..

[113] Ashrafmansouri M., Amiri-Dashatan N., Ahmadi N. (2022). Identification of protein profile in metacyclic and amastigote-like stages of *Leishmania tropica*: A proteomic approach. AMB Express.

[114] Biyani N., Madhubala R. (2012). Quantitative proteomic profiling of the promastigotes and the intracellular amastigotes of *Leishmania donovani* isolates identifies novel proteins having a role in *Leishmania* differentiation and intracellular survival. Biochim. Biophys. Acta.

[115] Bratkovic T., Rogelj B. (2014). The many faces of small nucleolar RNAs. Biochim. Biophys. Acta.

[116] van der Werf J., Chin C.V., Fleming N.I. (2021). SnoRNA in Cancer Progression, Metastasis and Immunotherapy Response. Biology.

[117] Huang Z.H., Du Y.P., Wen J.T., Lu B.F., Zhao Y. (2022). snoRNAs: Functions and mechanisms in biological processes, and roles in tumor pathophysiology. Cell Death Discov..

[118] Chikne V., Shanmugha Rajan K., Shalev-Benami M., Decker K., Cohen-Chalamish S., Madmoni H., Biswas V.K., Kumar Gupta S., Doniger T., Unger R. (2019). Small nucleolar RNAs controlling rRNA processing in *Trypanosoma brucei*. Nucleic Acids Res..

[119] Martinez-Calvillo S., Florencio-Martinez L.E., Nepomuceno-Mejia T. (2019). Nucleolar Structure and Function in Trypanosomatid Protozoa. Cells.

[120] Dupe A., Dumas C., Papadopoulou B. (2015). Differential Subcellular Localization of Leishmania Alba-Domain Proteins throughout the Parasite Development. PLoS ONE.

[121] Zhang X., Lai M., Chang W., Yu I., Ding K., Mrazek J., Ng H.L., Yang O.O., Maslov D.A., Zhou Z.H. (2016). Structures and stabilization of kinetoplastid-specific split rRNAs revealed by comparing leishmanial and human ribosomes. Nat. Commun..

[122] Dass S., Mather M.W., Ke H. (2020). Divergent Mitochondrial Ribosomes in Unicellular Parasitic Protozoans. Trends Parasitol..

[123] Gahura O., Chauhan P., Zikova A. (2022). Mechanisms and players of mitoribosomal biogenesis revealed in trypanosomatids. Trends Parasitol..

[124] Bieri P., Greber B.J., Ban N. (2018). High-resolution structures of mitochondrial ribosomes and their functional implications. Curr. Opin. Struct. Biol..

[125] Maslov D.A., Spremulli L.L., Sharma M.R., Bhargava K., Grasso D., Falick A.M., Agrawal R.K., Parker C.E., Simpson L. (2007). Proteomics and electron microscopic characterization of the unusual mitochondrial ribosome-related 45S complex in *Leishmania tarentolae*. Mol. Biochem. Parasitol..

[126] Ridlon L., Skodova I., Pan S., Lukes J., Maslov D.A. (2013). The importance of the 45 S ribosomal small subunit-related complex for mitochondrial translation in *Trypanosoma brucei*. J. Biol. Chem..

[127] Aphasizheva I., Maslov D.A., Aphasizhev R. (2013). Kinetoplast DNA-encoded ribosomal protein S12: A possible functional link between mitochondrial RNA editing and translation in *Trypanosoma brucei*. RNA Biol..

[128] Brown S.V., Hosking P., Li J., Williams N. (2006). ATP synthase is responsible for maintaining mitochondrial membrane potential in bloodstream form *Trypanosoma brucei*. Eukaryot. Cell.

[129] Schnaufer A., Clark-Walker G.D., Steinberg A.G., Stuart K. (2005). The F1-ATP synthase complex in bloodstream stage trypanosomes has an unusual and essential function. EMBO J..

[130] Ginger M.L. (2006). Niche metabolism in parasitic protozoa. Philos. Trans. R. Soc. B Biol. Sci..

[131] Zhang X., Deitsch K.W., Kirkman L.A. (2021). The contribution of extrachromosomal DNA to genome plasticity in malaria parasites. Mol. Microbiol..

[132] Laffitte M.N., Leprohon P., Papadopoulou B., Ouellette M. (2016). Plasticity of the *Leishmania* genome leading to gene copy number variations and drug resistance. F1000Res.

[133] Santi A.M.M., Murta S.M.F. (2022). Impact of Genetic Diversity and Genome Plasticity of *Leishmania* spp. in Treatment and the Search for Novel Chemotherapeutic Targets. Front. Cell. Infect. Microbiol..

[134] Cruz-Saavedra L., Vallejo G.A., Guhl F., Messenger L.A., Ramirez J.D. (2020). Transcriptional remodeling during metacyclogenesis in *Trypanosoma cruzi* I. Virulence.

[135] Cruz-Saavedra L., Vallejo G.A., Guhl F., Ramirez J.D. (2020). Transcriptomic changes across the life cycle of *Trypanosoma cruzi* II. PeerJ.

[136] Fiebig M., Kelly S., Gluenz E. (2015). Comparative Life Cycle Transcriptomics Revises *Leishmania mexicana* Genome Annotation and Links a Chromosome Duplication with Parasitism of Vertebrates. PLoS Pathog..

[137] Vasquez J.J., Hon C.C., Vanselow J.T., Schlosser A., Siegel T.N. (2014). Comparative ribosome profiling reveals extensive translational complexity in different *Trypanosoma brucei* life cycle stages. Nucleic Acids Res..

[138] Atwood J.A., Weatherly D.B., Minning T.A., Bundy B., Cavola C., Opperdoes F.R., Orlando R., Tarleton R.L. (2005). The *Trypanosoma cruzi* proteome. Science.

[139] Amorim J.C., Batista M., da Cunha E.S., Lucena A.C.R., Lima C.V.P., Sousa K., Krieger M.A., Marchini F.K. (2017). Quantitative proteome and phosphoproteome analyses highlight the adherent population during *Trypanosoma cruzi* metacyclogenesis. Sci. Rep..

[140] Amiri-Dashatan N., Ahmadi N., Rezaei-Tavirani M., Koushki M. (2021). Identification of differential protein expression and putative drug target in metacyclic stage of *Leishmania major* and *Leishmania tropica*: A quantitative proteomics and computational view. Comp. Immunol. Microbiol. Infect. Dis..

[141] Wang Z.X., Zhou C.X., Elsheikha H.M., He S., Zhou D.H., Zhu X.Q. (2017). Proteomic Differences between Developmental Stages of *Toxoplasma gondii* Revealed by iTRAQ-Based Quantitative Proteomics. Front. Microbiol..

[142] Wilson D.M., Deutsch W.A., Kelley M.R. (1994). Drosophila ribosomal protein S3 contains an activity that cleaves DNA at apurinic/apyrimidinic sites. J. Biol. Chem..

[143] Graifer D., Malygin A., Zharkov D.O., Karpova G. (2014). Eukaryotic ribosomal protein S3: A constituent of translational machinery and an extraribosomal player in various cellular processes. Biochimie.

[144] Carvalho C.M., Santos A.A., Pires S.R., Rocha C.S., Saraiva D.I., Machado J.P., Mattos E.C., Fietto L.G., Fontes E.P. (2008). Regulated nuclear trafficking of rpL10A mediated by NIK1 represents a defense strategy of plant cells against virus. PLoS Pathog..

[145] Mazumder B., Poddar D., Basu A., Kour R., Verbovetskaya V., Barik S. (2014). Extraribosomal l13a is a specific innate immune factor for antiviral defense. J. Virol..

[146] Kuhn J.F., Tran E.J., Maxwell E.S. (2002). Archaeal ribosomal protein L7 is a functional homolog of the eukaryotic 15.5kD/Snu13p snoRNP core protein. Nucleic Acids Res..

[147] Warner J.R., McIntosh K.B. (2009). How common are extraribosomal functions of ribosomal proteins?. Mol. Cell.

[148] Lindstrom M.S. (2009). Emerging functions of ribosomal proteins in gene-specific transcription and translation. Biochem. Biophys. Res. Commun..

[149] Lu H., Zhu Y.F., Xiong J., Wang R., Jia Z. (2015). Potential extra-ribosomal functions of ribosomal proteins in *Saccharomyces cerevisiae*. Microbiol. Res..

[150] Wool I.G. (1996). Extraribosomal functions of ribosomal proteins. Trends Biochem. Sci..

[151] Mishra P.C., Kumar A., Sharma A. (2009). Analysis of small nucleolar RNAs reveals unique genetic features in malaria parasites. BMC Genom..

[152] Wang L., Ciganda M., Williams N. (2013). Defining the RNA-protein interactions in the trypanosome preribosomal complex. Eukaryot. Cell.

[153] Liang X., Hart K.J., Dong G., Siddiqui F.A., Sebastian A., Li X., Albert I., Miao J., Lindner S.E., Cui L. (2018). Puf3 participates in ribosomal biogenesis in malaria parasites. J. Cell Sci..

[154] Fricker R., Brogli R., Luidalepp H., Wyss L., Fasnacht M., Joss O., Zywicki M., Helm M., Schneider A., Cristodero M. (2019). A tRNA half modulates translation as stress response in *Trypanosoma brucei*. Nat. Commun..

[155] Rajan K.S., Chikne V., Decker K., Waldman Ben-Asher H., Michaeli S. (2019). Unique Aspects of rRNA Biogenesis in Trypanosomatids. Trends Parasitol..

[156] Nepomuceno-Mejia T., Florencio-Martinez L.E., Pineda-Garcia I., Martinez-Calvillo S. (2022). Identification of factors involved in ribosome assembly in the protozoan parasite Leishmania major. Acta Trop..

[157] Pircher A., Gebetsberger J., Polacek N. (2014). Ribosome-associated ncRNAs: An emerging class of translation regulators. RNA Biol..

[158] Mleczko A.M., Machtel P., Walkowiak M., Wasilewska A., Pietras P.J., Bakowska-Zywicka K. (2019). Levels of sdRNAs in cytoplasm and their association with ribosomes are dependent upon stress conditions but independent from snoRNA expression. Sci. Rep..

[159] Dumas C., Chow C., Muller M., Papadopoulou B. (2006). A novel class of developmentally regulated noncoding RNAs in Leishmania. Eukaryot. Cell.

[160] Rajan K.S., Doniger T., Cohen-Chalamish S., Rengaraj P., Galili B., Aryal S., Unger R., Tschudi C., Michaeli S. (2020). Developmentally Regulated Novel Non-coding Anti-sense Regulators of mRNA Translation in *Trypanosoma brucei*. iScience.

[161] Guegan F., Rajan K.S., Bento F., Pinto-Neves D., Sequeira M., Guminska N., Mroczek S., Dziembowski A., Cohen-Chalamish S., Doniger T. (2022). A long noncoding RNA promotes parasite differentiation in African trypanosomes. Sci. Adv..

[162] Leroux L.P., Lorent J., Graber T.E., Chaparro V., Masvidal L., Aguirre M., Fonseca B.D., van Kempen L.C., Alain T., Larsson O. (2018). The Protozoan Parasite *Toxoplasma gondii* Selectively Reprograms the Host Cell Translatome. Infect. Immun..

[163] Vesper O., Amitai S., Belitsky M., Byrgazov K., Kaberdina A.C., Engelberg-Kulka H., Moll I. (2011). Selective translation of leaderless mRNAs by specialized ribosomes generated by MazF in *Escherichia coli*. Cell.

[164] Kurylo C.M., Parks M.M., Juette M.F., Zinshteyn B., Altman R.B., Thibado J.K., Vincent C.T., Blanchard S.C. (2018). Endogenous rRNA Sequence Variation Can Regulate Stress Response Gene Expression and Phenotype. Cell Rep..

[165] Lopez-Lopez A., Benlloch S., Bonfa M., Rodriguez-Valera F., Mira A. (2007). Intragenomic 16S rDNA divergence in *Haloarcula marismortui* is an adaptation to different temperatures. J. Mol. Evol..

[166] Chavez-Fumagalli M.A., Costa M.A., Oliveira D.M., Ramirez L., Costa L.E., Duarte M.C., Martins V.T., Oliveira J.S., Olortegi C.C., Bonay P. (2010). Vaccination with the *Leishmania infantum* ribosomal proteins induces protection in BALB/c mice against Leishmania chagasi and Leishmania amazonensis challenge. Microbes Infect..

[167] Ramirez L., Santos D.M., Souza A.P., Coelho E.A., Barral A., Alonso C., Escutia M.R., Bonay P., de Oliveira C.I., Soto M. (2013). Evaluation of immune responses and analysis of the effect of vaccination of the *Leishmania major* recombinant ribosomal proteins L3 or L5 in two different murine models of cutaneous leishmaniasis. Vaccine.

[168] Prava J., Pan A. (2022). In silico analysis of Leishmania proteomes and protein-protein interaction network: Prioritizing therapeutic targets and drugs for repurposing to treat leishmaniasis. Acta Trop..

[169] Clayton C.E. (2002). Life without transcriptional control? From fly to man and back again. EMBO J..

[170] Smircich P., Eastman G., Bispo S., Duhagon M.A., Guerra-Slompo E.P., Garat B., Goldenberg S., Munroe D.J., Dallagiovanna B., Holetz F. (2015). Ribosome profiling reveals translation control as a key mechanism generating differential gene expression in *Trypanosoma cruzi*. BMC Genom..

